# Assessment of sensory and nutritional attributes of foxtail millet-based food products

**DOI:** 10.3389/fnut.2023.1146545

**Published:** 2023-04-17

**Authors:** Laghima Arora, Renuka Aggarwal, Inderpreet Dhaliwal, Om Prakash Gupta, Prashant Kaushik

**Affiliations:** ^1^Department of Food Science and Technology, Punjab Agricultural University, Ludhiana, Punjab, India; ^2^Department of Plant Breeding and Genetics, Punjab Agricultural University, Ludhiana, Punjab, India; ^3^ICAR-Indian Institute of Wheat and Barley Research, Karnal, Haryana, India; ^4^Instituto de Conservación y Mejora de la Agrodiversidad Valenciana, Universitat Politècnica de València, Valencia, Spain

**Keywords:** foxtail millet, value-added products, protein, resistant starch, predicted glycemic index

## Abstract

Millets are a rich source of many health-promoting nutrients as well as bioactive compounds such as dietary fibers, antioxidants, macro and micronutrients etc., compared to other staple cereals such as rice, wheat and maize. These nutrients play a central role in the world nutritional security. Despite the inbuilt nutritional benefits, the production of millets has witnessed sharp decline owing to taste preferences, keeping quality and challenges associated with food preparation from millets. To sensitize the consumers about the nutritional benefits of foxtail millet, the present study was planned to formulate and nutritionally evaluate eight diversified foxtail millet-based food products namely rusk, *kheer*, *pinni*, *sattu*, vegetable *dalia*, cookies, bar and *papad* by replacing commonly used cereals such as wheat and rice. The products prepared from Foxtail millet were found to have high acceptability with mean score of more than 8.00. These diversified food products showed higher protein content ranging from 10.98 to 16.10 g/100 g, with the highest protein found in Foxtail millet kheer (16.01 g/100 g). The resistant starch content and predicted glycemic index (PGI) of these products ranged between 13.67 to 22.61 g/100 g and 46.12 to 57.55, respectively, with the highest resistant starch (22.61 ± 0.69 g/100 g) and lowest PGI (48.42 ± 0.20) found in millet bar. The high resistant starch and low PGI in foxtail millet products suggest that they could serve as an excellent food source suitable for diabetics. The obtained results suggest that all the Foxtail millet-based value-added products have superior nutrient profile and are highly acceptable than the traditional products. Inclusion of these foods in the diets of the population may help in the prevention of malnutrition and type 2 diabetes.

## 1. Introduction

With 109.52 and 122.27 million tonnes of production in 2021 (1), India is the world’s second largest producer of wheat and rice, respectively (2). The cultivation of staple grains like wheat and rice in the Indo Gangetic Plains significantly contributed to the green revolution and helped to ensure the country’s food security. However, unbalanced fertilizer application, decreased soil fertility owing to nutrient depletion, decreased recycling of soil and water resources, anomalies in the pH of the soil and water balance, a rise in disease and rodent incidence, and the destruction of natural ecosystems are all variables that have a detrimental impact on the production systems’ ability to sustain itself (3). Therefore, farming practices must be diversified such that they are both environmentally sustainable and nutritionally appropriate. Crop diversification is required not only to address the issues arising from wheat-rice farming system, but also to address the situation of globally rising non-communicable diseases (NCDs) and malnutrition. NCDs are caused by an interplay of biological systems with dietary variables, such as a lack of nutrient-rich functional foods and an abundance of quickly absorbed energy-rich foods (4). People’s lifestyles and eating habits have been significantly altered by globalization and industrialization, which have led to weakened immunity and thus leading to non-communicable diseases (5). The rising trend of refined cereal intake aggravates the health situation further, leading to overnutrition and NCDs among different population groups worldwide.

Millets, also known as Nutri-cereals, are a type of grasses with small seeds. These were a staple diet in many countries decades ago, but are now despised as “minor grains.” They are grown all over the world as cereal grains for human and animal nutrition. However, both their production and consumption have experienced a significant fall. Poor wages, a lack of input subsidies and price incentives, and the subsidized supply of fine cereals through the public distribution system are the main causes of the decline. Additionally, a lack of understanding of the nutritional benefits, challenges in food preparation, and a lack of processing technology are some additional significant issues that have contributed to millets becoming more obsolete (6).

Many nutrients, dietary fiber, and antioxidants are deficient in diets based entirely on wheat and rice (7, 8). Compared to staple cereals, millets are nutritionally dense and encompass several health-promoting functional compounds (9). They contain high concentrations of macro and micronutrients such as proteins, dietary fiber, carbohydrates, phytochemicals and vital amino acids (10, 11). Millets have a balanced nutritional profile, and consuming them has been shown to have positive effects on health outcomes. Millets have the potential to promote diversification as well as provide food and nutrition security. Millets have a huge potential to work as alternative grains for ensuring food and nutritional security in most parts of the world, due to their high concentrations of micronutrients, dietary fiber, vitamins, and phytochemicals with diverse therapeutic applications (12). It was stated that the presence of high amounts of polyphenols and other bioactive compounds in millets increase the rate of fat absorption and the time of release of sugars, thereby decreasing the glycemic index (13). The quality as well as quantity of dietary fiber and resistant starch in the millet grain is better than the other cereals. These components aid to provide a feeling of prolong fullness and prevention of constipation. Moreover, due to the presence of tryptophan, consumption of millets leads to more production of serotonin, causing a soothing effect on human mind. It was reported that millet’s fiber binds to toxins in stomach and may help in the protection against colon cancer (14). Another study reported that protein extracted from foxtail millet bran exhibits anti-colon cancer properties and may be used as a therapeutic treatment for colon cancer (15).

A study conducted on type 2 diabetic patients by supplementing their diet with millets. The results showed that the supplementary diet improved the levels of antioxidant, vitamins, calcium, magnesium and hemoglobin (16). The HbA1C, blood sugar, blood pressure and pro inflammatory cytokines were also found to be decreased with the millet-based diet. Similarly, it was reported that millet exhibit hypolipidemic effect by regulating lipid metabolism related genes expression or gut microbiota composition (17).

Due to the health-promoting properties of millets, production and development of millet-based products is gaining support in different parts of the world. The introduction of new technology for the production of alternative foods, commercial goods, and animal feed has the potential to increase millet’s demand. They can be used in both traditional and innovative foods. Millets have been successfully used to make cakes, cookies, pasta, parboiled rice- like products, and snack foods (18). Foxtail millet was used to create an unfortified weaning mix with acceptable sensory and rheological properties (19), a composite bread having low glycemic index (20), ready-to-eat extruded snack (21), biscuits and burfi with low glycemic index (22), and beverages with high anti-oxidant activity (23). Foxtail millet (*Setaria italica*) is a minor millet that contains 11% protein, 59.1% starch, 3.9% fat, 19.1% dietary fiber, 7.0% ash and 6.6 mg/100 g of phenolic compounds (24). There is availability of huge literature on the millets like Pearl millet and Sorghum but less emphasis has been given on Foxtail millet. So, the current investigation was focussed on development and nutritional analysis of Foxtail millet-based food products, so as to provide a healthier dietary option to the vulnerable population.

## 2. Materials and methods

### 2.1. Procurement of sample

**Figure 1:**
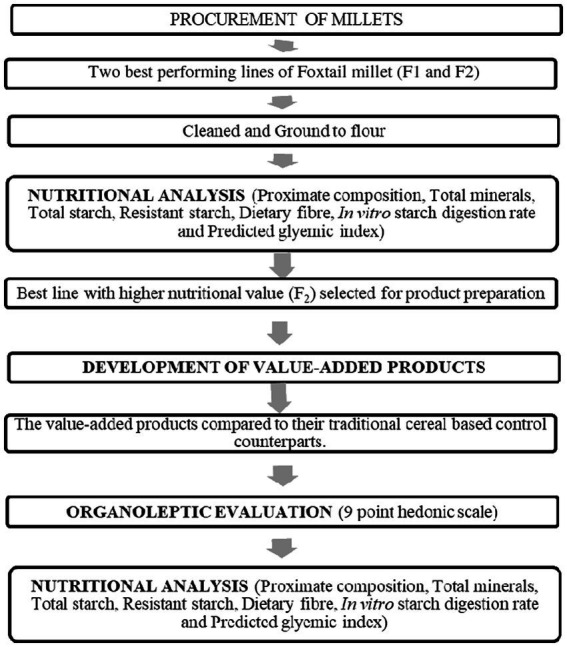
Methodology of the study.

After a thorough screening of a germplasm set of 1,000 lines across 4 years, two short-duration (60–65 days) lines of Foxtail Millet (), which were moderate in yield, were shortlisted for multilocation trials across Punjab in the summer season and were considered for this study. These were namely F1 and F2, respectively, and were procured from the Department of Plant Breeding and Genetics, College of Agriculture, PAU, Ludhiana. The procured seeds were cleaned to remove any impurity and the samples were stored in air-tight containers for nutritional analysis and product development (Figure 1). The cereal grains like wheat, rice, barley and other ingredients required for the development of food products were procured from the local market.

### 2.2. Chemical analysis

The cleaned raw grains of the selected germplasm lines were ground to a flour using an electric grinder with 20 mesh size and was stored at ambient conditions for further nutritional analysis. The flour samples were analyzed in triplicates for proximate composition, minerals (calcium, iron, zinc), resistant starch, dietary fiber, *in vitro* starch digestion rate and predicted glycemic index, using the standard operating procedures described below.

### 2.3. Determination of proximate composition

Proximate parameters namely moisture, crude protein, crude fat, crude fiber and ash were analyzed using three replications of each raw germplasm line using the standard method given by (25). For moisture determination, 5 g of sample was weighed and placed in pre-weighed china crucibles in a hot air oven at 105°C for 8 h to dry to a constant weight. For the determination of nitrogen, the macro-kjeldahl method was used. Nitrogen was converted to crude protein using a conversion factor of 6.25. The crude fat content was determined using soxhlet assembly, where moisture-free sample was put in a thimble. Petroleum ether was used as a solvent. The extracted fat from the sample was weighed after evaporating the remaining solvent. 5 g of (moisture- and fat- free) sample was mixed with 200 ml of 1.25% H2SO4. A Buchner funnel was used to filter the sample through a cotton cloth after it had been refluxed for 30 min for the estimation of crude fat. The residue was cleaned with hot water until it was acid-free. The residue was mixed with 200 ml of 1.25% NaOH. It was refluxed for another 30 min before being filtered through muslin cloth. It was rinsed in hot water once again. The residue was transferred to the previously weighed crucible and dried for 2 h at 130°C in a hot air oven. After being ignited in a muffle furnace and cooled in a desiccator, the weight loss was estimated. The ash content was analyzed using 0.5 g of sample which was placed in previously weighed and labeled crucibles. The samples were heated to 550°C in a muffle furnace for 4 h. After cooling in a desiccator, the crucible residue was weighed again. The carbohydrate content was calculated by subtracting sum of all proximate parameters (moisture content, crude protein, crude fat, crude fiber, and total ash) from 100.

### 2.4. Mineral analysis

The powdered flour was digested by the addition of a triple acid combination made up of nitric acid (HNO3), sulfuric acid (H2SO4), and perchloric acid (HClO4), in the ratio of 10:4:1in order to measure iron, zinc and calcium. Following digestion, the samples were diluted and filtered, and the volume was made to 100 ml. Following the initial preparations, the amount of iron, zinc and calcium was measured by comparing with standards for these elements using an atomic absorption spectrophotometer (Analyst 200, Perkin Elmer).

### 2.5. Dietary fiber

The soluble, insoluble and total dietary fiber contents of the samples were analyzed in triplicates using Megazyme-K-TDFR-200A. The soluble and insoluble dietary fiber contents were analyzed using the standard protocol given by (25). The dietary fiber was calculated using the formula:

$$\mathrm{Dietary fiber}\;\left(\%\right)=\frac{\frac{R_{1}+R_{2}}{2}-p-A-B}{\frac{m_{1+m_{2}}}{2}}\times 100$$

Where:

R_1_ = residue weight 1 from m1, R_2_ = residue weight 2 from m_2_, m_1_ = sample weight 1,

m_2_ = sample weight 2, A = ash weight from R_1_,

p = protein weight from R_2_ and

B = blank = $\frac{BR_{1}+BR_{2}}{2}-BP-BA$

Where:

BR = blank residue,

BP = blank protein from BR1, BA = blank ash from BR2.

### 2.6. Total starch and resistant starch

The starch content was analyzed using method given by (26). Megazyme K-RSTAR assay kit was used for the estimation. Resistant starch and non-resistant (solubilized) starch were analyzed and the total amount of starch was determined by the addition of these two components.

### 2.7. *In vitro* starch digestion rate

Five hundred mg of the sample was treated for 15–20 s with 1 ml of artificial saliva containing porcine -amylase (Sigma A-3176 Type VI-B; 250 U per ml of carbonate buffer) before 5 ml of pepsin (Sigma P-6887, from gastric porcine mucosa; 1 ml per ml of 0.02 M aqueous Hydrochloric acid) was added and incubated at 37°C for 30 min in a shaking water bath. The digesta was neutralized (5 ml of 0.02 M aq. Sodium hydroxide) before the pH was set to 6 (25 ml of 0.2 M sodium acetate buffer) before adding 5 ml of pancreatin (Sigma P1750 from porcine pancreas; 2 mg per ml of acetate buffer) and amyloglucosidase (Sigma A- 7420 from *Aspergillus niger*; 28 U per ml of acetate buffer). The solution was incubated for 4 h, during which time the glucose concentration in the digesta was monitored using an AccuCheck^®^ Performa^®^ glucometer at various intervals.

### 2.8. Rapidly digestible starch and slowly digestible starch

The glucometer reading at 15 min was converted to the percentage of starch digested using the following equation

Where:

$$DS=\frac{0.9\times G_{G}\times 180\times V}{W\times S\left[100-M\right]}$$

GG = Reading of the glucometer (mM/L)

V = Digest volume (mL),

180 = glucose’s molecular weight W = sample weight (g)

S = sample’s starch content (g per 100 g dry sample)

M = moisture percentage in sample (g per 100 g sample)

0.9 = starch stoichiometric constant from glucose concentrations.

RDS% = percentage of starch digested at 15 min

SDS% = percentage of starch digested at 120 min – percentage of starch digested at 15 min.

### 2.9. Predicted glycemic index

Predicted glycemic index was calculated using the method given by (27). For calculating predicted glycemic index, Hydrolysis index was worked out as:

$$HI=\frac{\mathrm{Area under hydrolysis curve of the test sample}}{\mathrm{Area under control}\;\left(\mathrm{white bread}\right)}$$

Then, the estimated glycemic index was calculated by applying the formula defined by (27): GI = 39.71 + 0.549 HI.

Glycemic load was calculated as:

$$\mathrm{Glycemic load}=\frac{\mathrm{GI}\times \mathrm{Available carbohydrates}}{100}$$

## 3. Development of foxtail millet- based food products

The nutritionally superior germplasm line of Foxtail millet (F2) was used for the preparation of value-added products, which were compared with the recipes prepared using traditional cereal [wheat for *rusk*, cookies, vegetable *dalia* and *pinni*, rice for *papad* and *kheer* (refrigerated) and barley for *sattu*] and was treated as control. Initially, millets were replaced with a traditional cereal (wheat, rice, and barley) at the level of 10%; however, all the products of Foxtail millet were found to be acceptable even at 100% replacement. Therefore, the recipes prepared after replacing 100% of wheat, rice and barley rice were developed and analyzed. The ingredients and procedure used to develop the value-added products are given in Table 1. Eight diversified foxtail millet-based products were developed in the category of traditional products including:

1. *kheer* (form of porridge prepared from milk and rice/millet);
2. *pinni* (mixture of roasted cereal/millet flour rolled into balls after adding *ghee* and sugar); baked products – *rusk* and cookies;
3. breakfast cereals – vegetable *dalia* (porridge); and snack foods – *sattu* (traditional drink), bar and *papad*.

**Table 1:** Value- added products developed using foxtail millet.

Ingredients	Traditional cereal- based products	Foxtail millet- based products	Procedure
Rusk	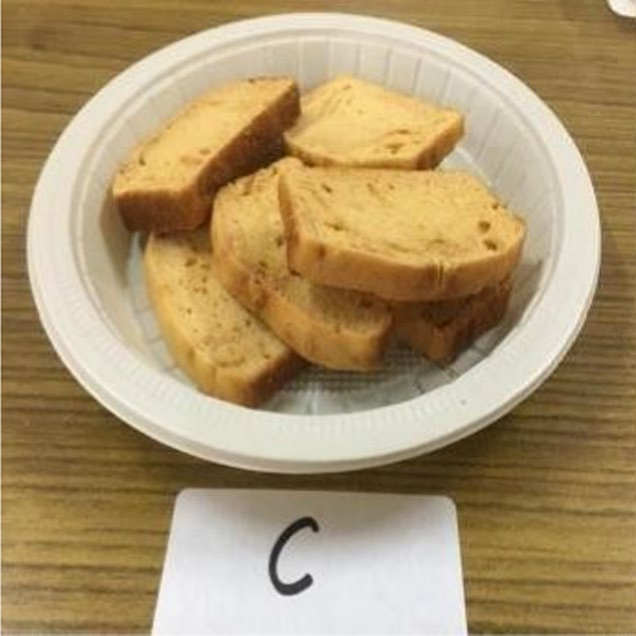	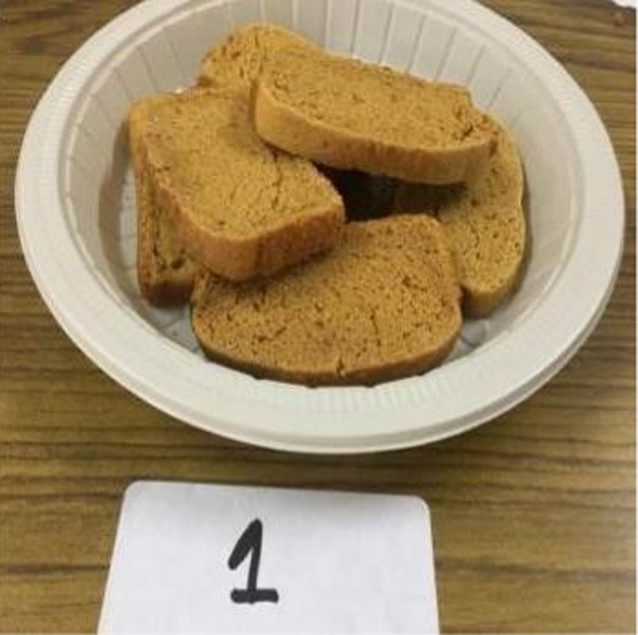	All the dry ingredients were sieved together and mixed with milk and baked at 180°C for 30 min to make a cake. Cake was cooled and sliced, then again baked at 130°C for 10 min to make rusk.
Refined wheat flour	200 gram	–	
Foxtail millet flour	–	200 gram	
Sugar	80 gram	80 gram	
Milk powder	50 gram	50 gram	
Baking powder	1 tsp	1 tsp	
Baking soda	1/2 tsp	1/2 tsp	
Milk	250 ml	250 ml	
			In a pan washed rice/ millet was added to milk and boiled on a very slow flame for atleast 1 h. Sugar was added and kheer was cooked for another 10 min. Cardamom powder added toward the end and *kheer* was garnished with dry fruits (28).
*Kheer*	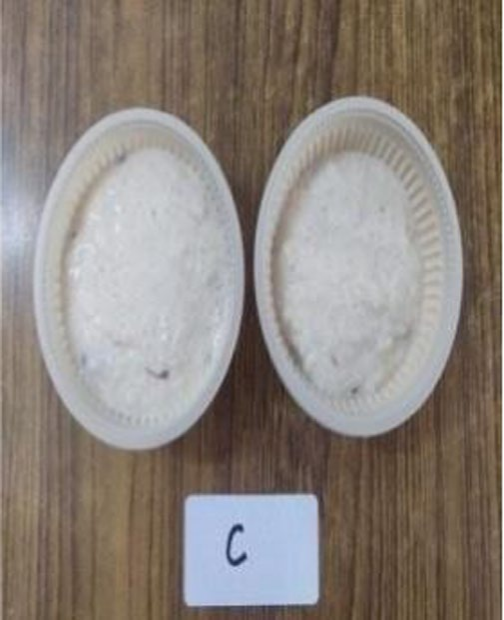	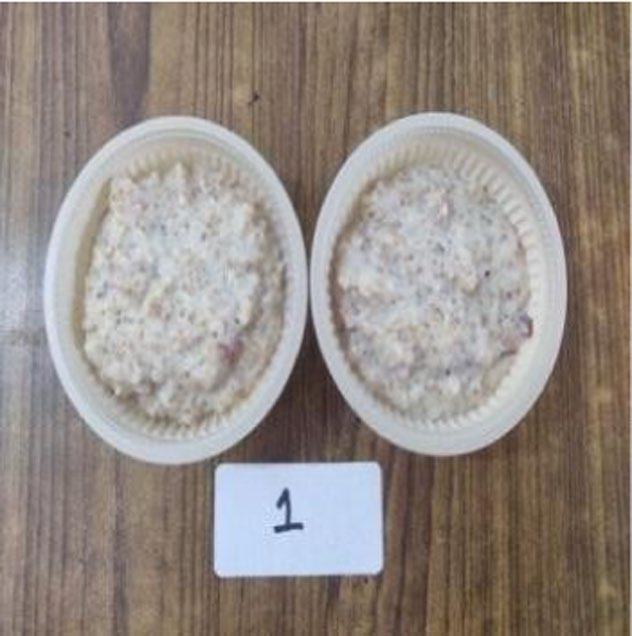	
Rice	100 gram	–	
Foxtail millet	–	100 gram	
Sugar	75 gram	75 gram	
Milk	1 liter	1 liter	
Cardamom powder	1/8 tsp	1/8 tsp	
Dry fruits	For garnishing	For garnishing	
			Millet flour/ whole wheat flour was roasted in ghee till aromatic. Dry roasted flaxseed powder was mixed with shakkar and added to the roasted flour after switching off the flame. *Pinni* was prepared from the hot mixture (29).
*Pinni*	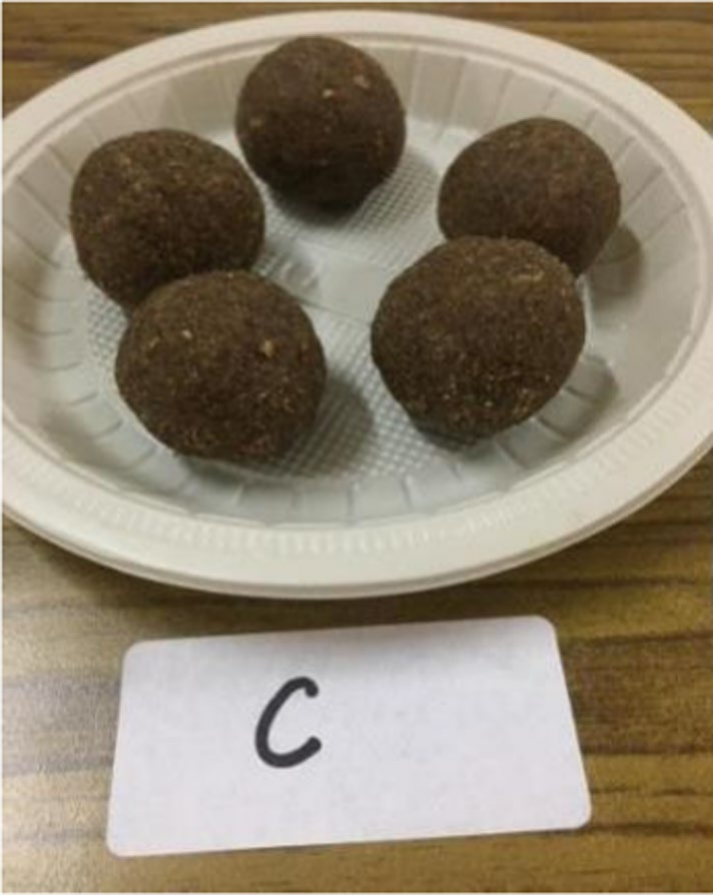	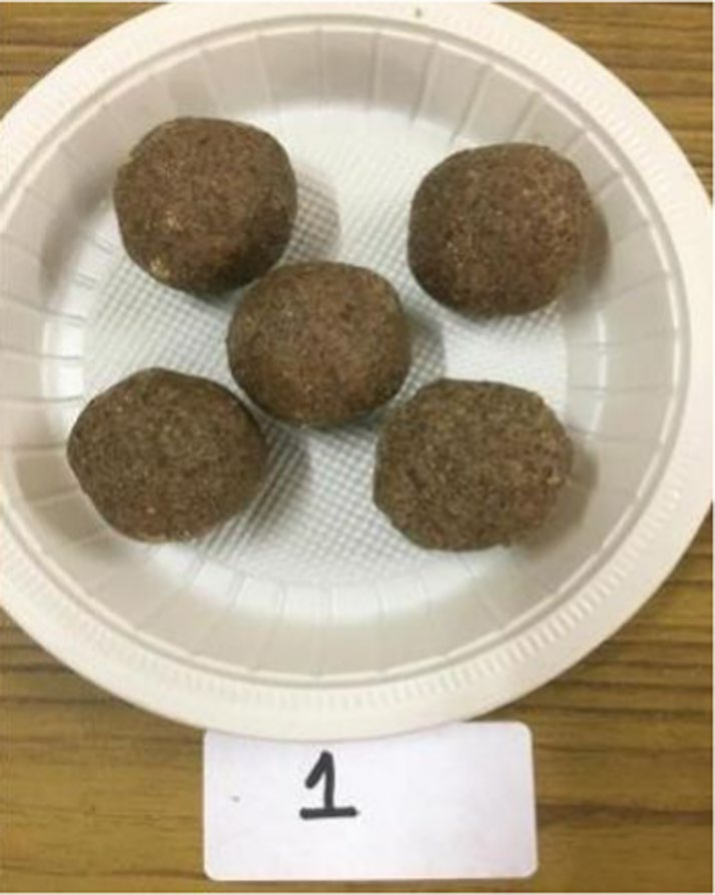	
Whole wheat flour	100 gram	–	
Foxtail millet flour	–	100 gram	
Roasted and powdered Flaxseeds	100 gram	100 gram	
Shakkar	100 gram	100 gram	
Desi ghee	100 gram	100 gram	
			The grains were roasted on a very low flame for 50–60 min or till the grains turned aromatic. The grains were cooled and ground into a fine powder. For making drink, 2 tsp. of this *sattu* powder was added to 250 ml water. Flavors like mint leaves, lemon, *shakker* and salt can also be added to drink (30).
*Sattu*	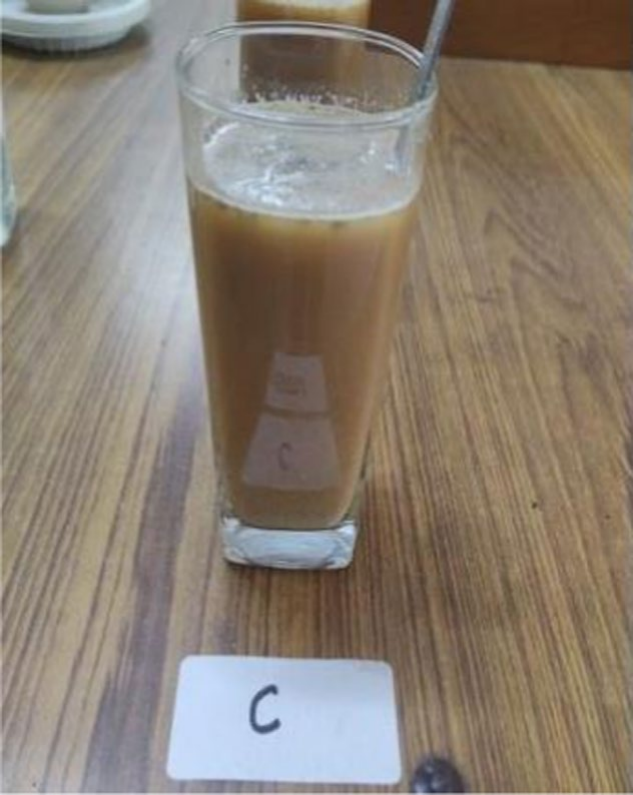	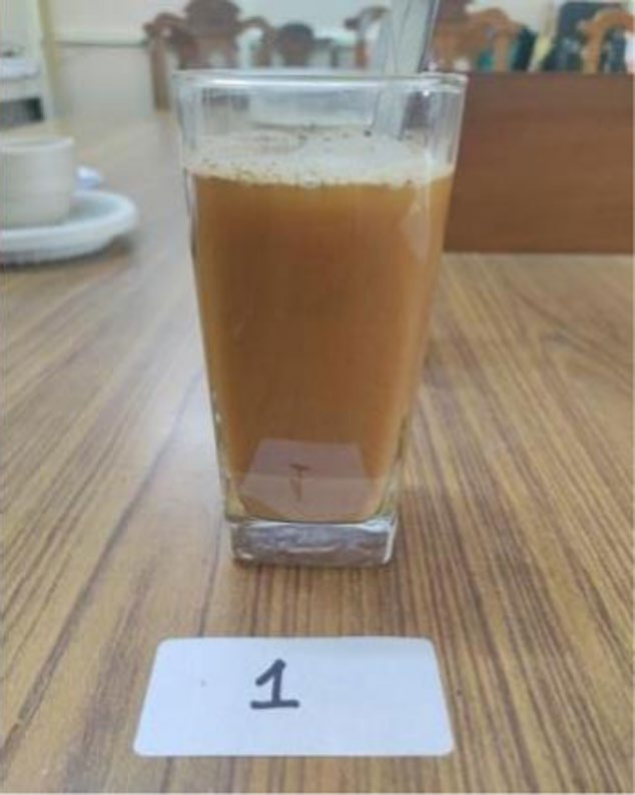	
Barley grains	100 gram	–	
Foxtail millet grains	–	100 gram	
			All the vegetables were sauteed in oil. Spices were added when vegetables were half done. Soaked millet (soaked for 2 h) or wheat *dalia* (without soaking) was added along with 200 ml of water. *Dalia* was pressure cooked till tender (31).
Vegetable *Dalia*	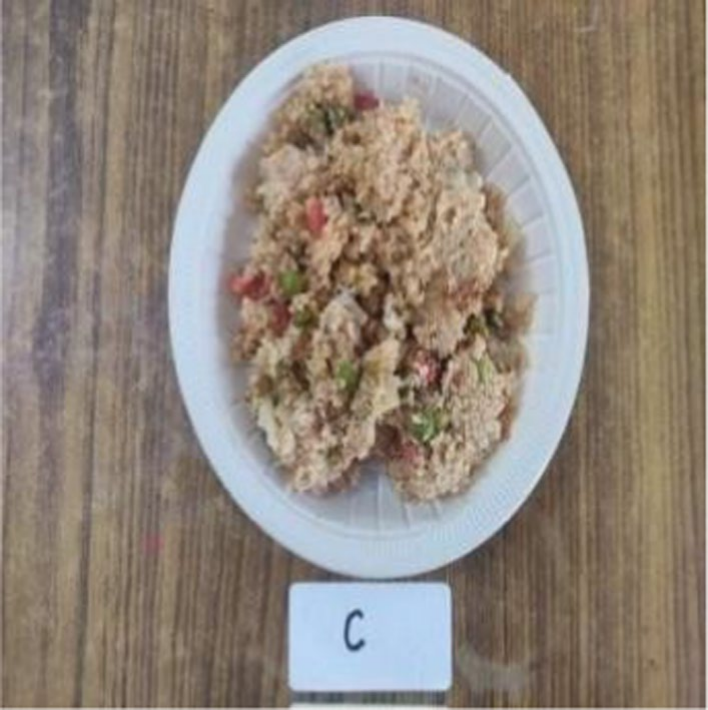	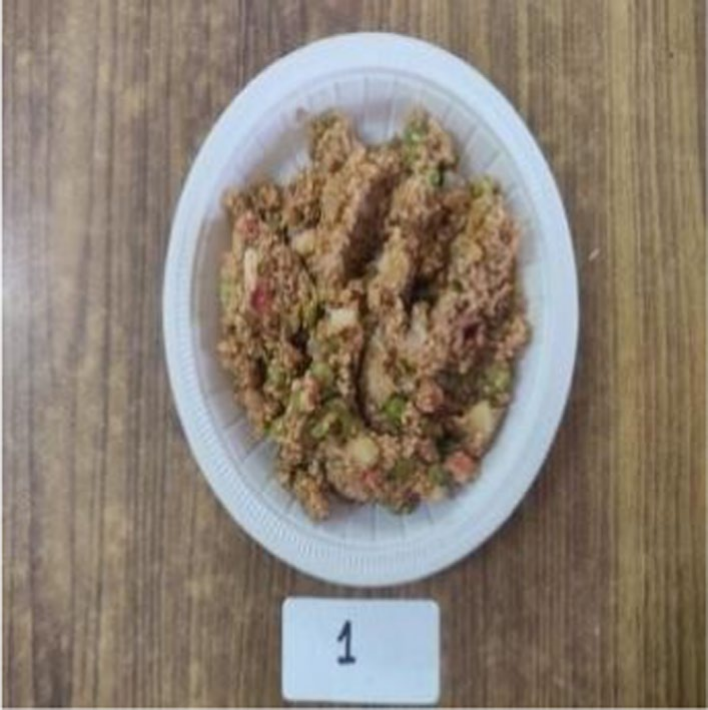	
Broken wheat	100 gram	–	
Foxtail millet	–	100 gram	
Onion	15gram	15gram	
Carrot	15gram	15gram	
Peas	15gram	15gram	
Cauliflower	15gram	15gram	
Green chilies	2 in no.	2 in no.	
Salt	1 tsp	1 tsp	
Red chili powder	1/4 tsp	1/4 tsp	
*Dhania* powder	1/2 tsp	1/2 tsp	
Oil	2 tsp	2 tsp	
			Butter and sugar were creamed and all the sieved dry ingredients were added to the creamed mixture. The mixture was formed into a dough and refrigerated for 15 min. The dough was shaped into cookies and baked in a preheated oven at 190°C for 10–12 min or till the edges turn brown. Cookies were cooled completely before serving (32).
Cookies	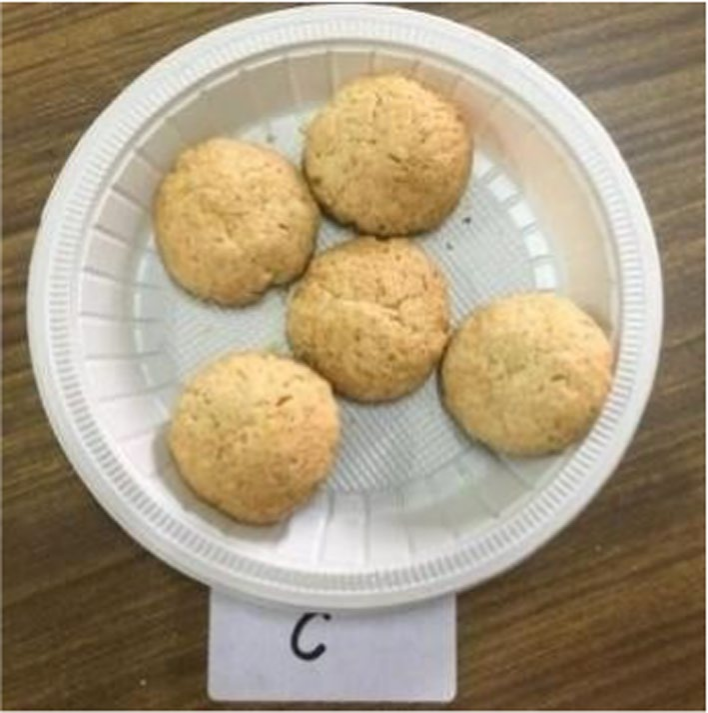	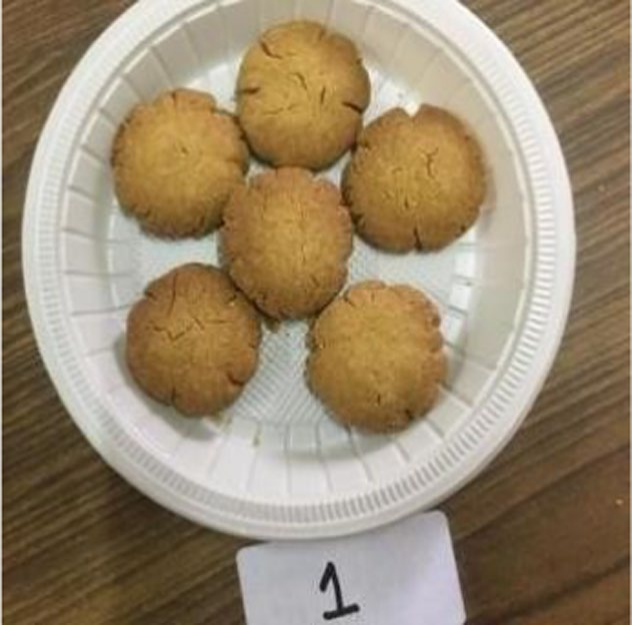	
Refined wheat flour	150 gram	–	
Foxtail millet flour	–	150 gram	
Butter	100 gram	100 gram	
Powdered sugar	100 gram	100 gram	
Baking powder	1 tsp	1 tsp	
Baking soda	1/4 tsp	1/4 tsp	
Cardamom powder	1/8 tsp	1/8 tsp	
Milk	20 ml	20 ml	
			All the ingredients were combined and a batter of thick consistency was prepared. Round *papad* were made with the help of a spatula on a pre greased plate. The *papad* were steamed for 10 min and dried in sun for 2 days or in a hot air oven at 100°C for 45 min. For serving, prepared *papad, it can* be fried as well as roasted in a microwave (33).
*Papad*	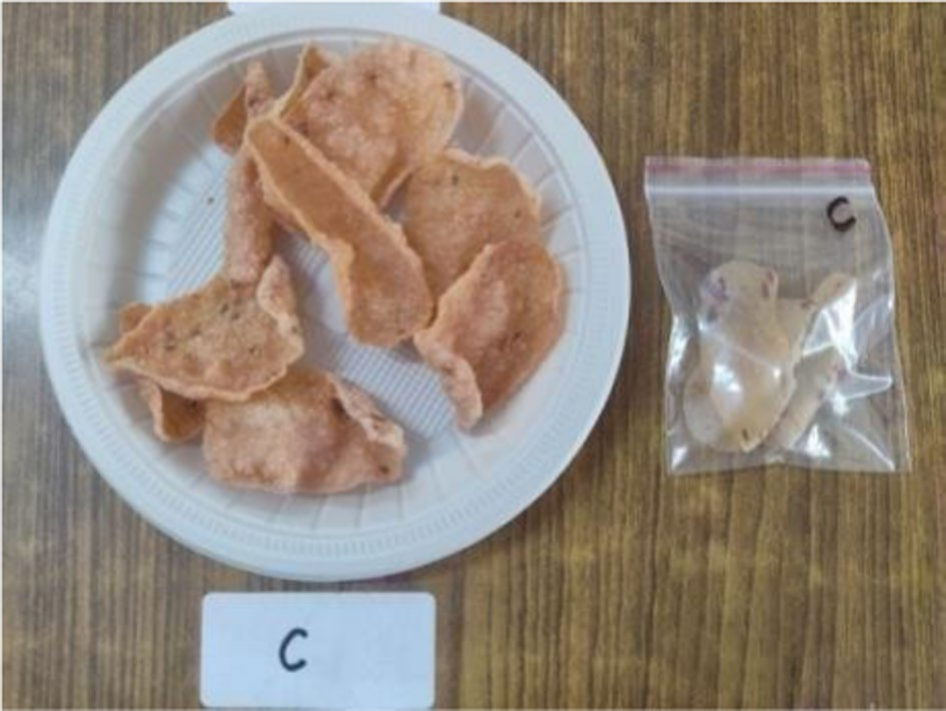	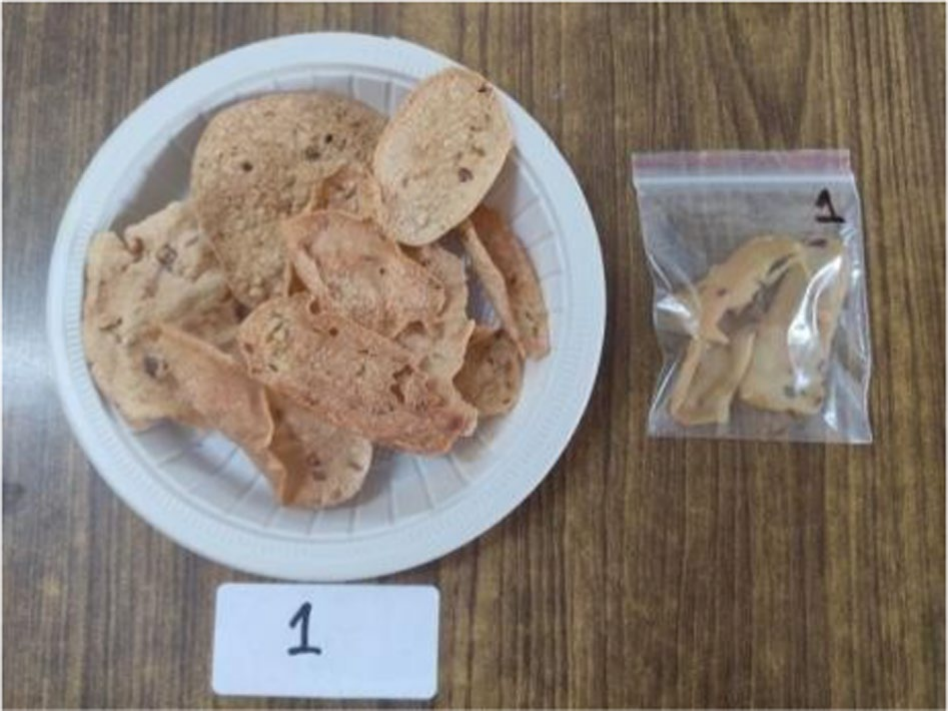	
Rice flour	100 gram	–	
Foxtail millet flour	–	100 gram	
Salt	1 tsp	1 tsp	
Black pepper	1/2 tsp	1/2 tsp	
Chili flakes	1/2 tsp	1/2 tsp	
Water	As required for achieving consistency	As required for achieving consistency	
Bar	There was no control for millet bar as there is no traditional recipe available.	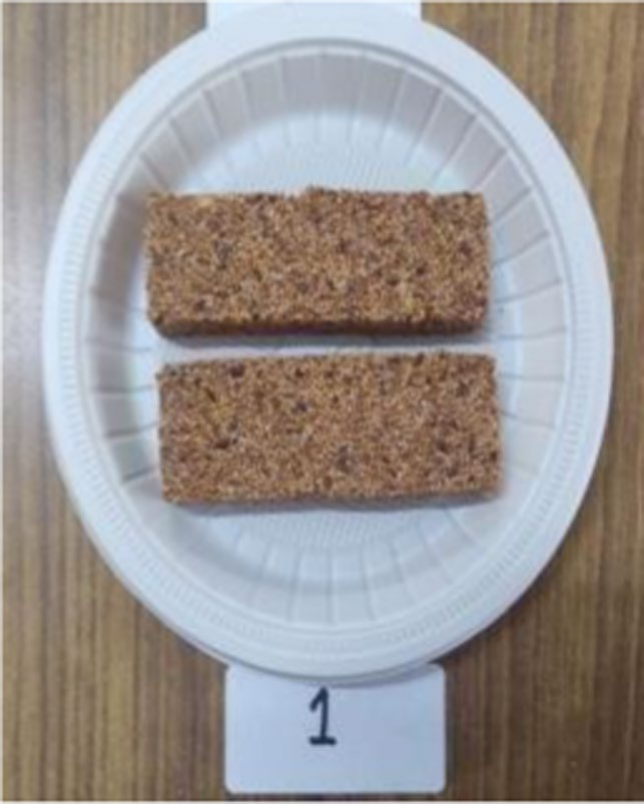	The millet seeds, peanuts and flaxseeds were roasted separately. Once cooled the seeds were coarsely ground. He seeds and honey were mixed together. The mixture was set in pre greased molds of size 12X3X2 cm. The bars were refrigerated for setting (33).
Foxtail millet		60 gram	
Peanuts		12.5 gram	
Flaxseed		12.5 gram	
Honey		15 gram	

### 3.1. Organoleptic evaluation of developed food products

The developed products were evaluated by a panel of minimum semi-trained individuals from the Department of Food and Nutrition, Punjab Agricultural University, Ludhiana for their sensory qualities. The panelists scored different products prepared from Foxtail millet based on their appearance, texture, taste, flavor and overall acceptability by using nine-point Hedonic rating scale (34), where 9 indicated “like extremely” and 1 indicated “dislike extremely.”

### 3.2. Nutritional evaluation of developed food products

The products prepared from Foxtail millet along with control prepared from traditional cereal (wheat/ rice) were analyzed for moisture, protein, fat, crude fiber, calcium, iron, zinc, starch, resistant starch, dietary fiber, *in vitro* starch digestion rate, predicted glycemic index using the same methods described above.

### 3.3. Statistical analysis of data

The data was analyzed in a completely randomized design using SPSS software (26 version). Mean and standard deviation for the various parameters were computed. Analysis of Variance (One-way ANOVA) was employed to assess the sensory and nutritional parameters of the raw Foxtail millet and for comparing the value-added products prepared from Foxtail millet.

## 4. Results

### 4.1. Proximate composition of the raw millets

**Figure 2:**
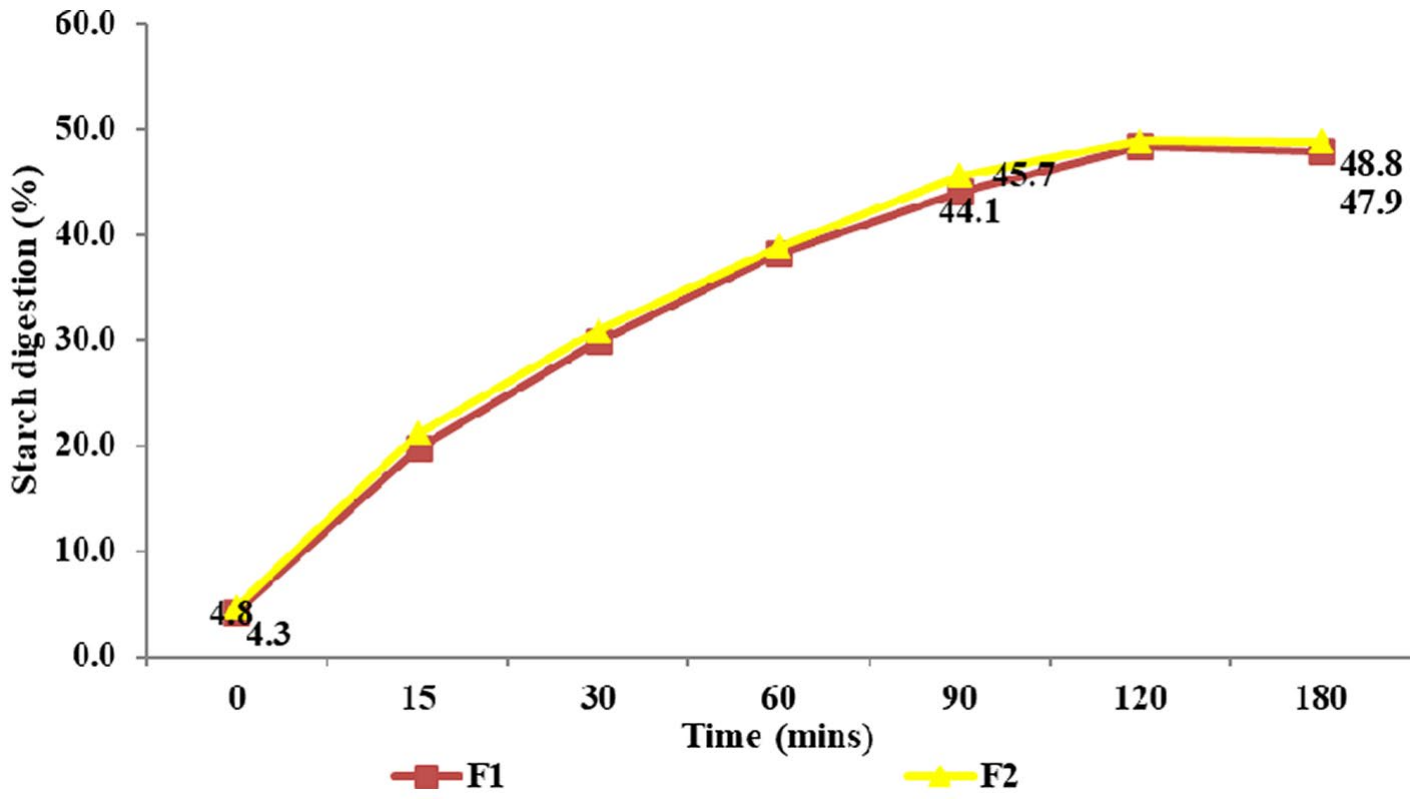
*In vitro* starch digestion rate of Foxtail millet.

The nutritional analysis of the two germplasm lines was done to find out the difference between their nutritional profile and to select the best line for the development of value-added products. The nutritional composition showed that germplasm line F2 was better than F1, as it had higher protein (13.59 ± 0.06) g, fat (4.18 ± 0.07) g, iron (4.53 ± 0.07) mg, calcium (54.85 ± 0.04) mg, zinc (3.44 ± 0.03) mg, total dietary fiber (14.06 ± 0.04) g and resistant starch contents (27.01 ± 0.59) g per 100 g on dry weight basis (Table 2). The germplasm line F2 also showed a low predicted glycemic index of 48.94 ± 0.21, with a glycemic load of 28.83 ± 0.46. The *in vitro* starch digestion rate of both germplasm lines of Foxtail millet is shown in Figure 2. F1 had a slower digestion rate of 47.9% compared to 48.8% in F2. The nutritional composition of the germplasm lines thus indicated that the line F2 contained higher amounts of protein, calcium, total dietary fiber and resistant starch along with lower predicted glycemic index and glycemic load compared to F1 (Table 2) and therefore, it was selected for the development of value-added products.

**Table 2:** Nutritional composition of Foxtail millet germplasm lines (on dry weight basis).

Parameter	F1	F2
Proximate composition		
Moisture (%)	10.82 ± 0.04^b^	9.94 ± 0.11^a^
Protein (g/100 g)	12.38 ± 0.68^a^	13.59 ± 0.06^b^
Crude fat (g/100 g)	3.35 ± 0.09^a^	4.18 ± 0.07^b^
Crude fiber (g/100 g)	7.06 ± 0.12^a^	7.83 ± 0.07^a^
Ash (g/100 g)	3.77 ± 0.21^a^	4.2 ± 0.10^b^
CHO (g/100 g)	58.91 ± 0.72^a^	60.27 ± 0.24^b^
Mineral composition		
Iron (mg/100 g)	4.22 ± 0.03^a^	4.53 ± 0.07^b^
Calcium (mg/100 g)	53.25 ± 0.04^a^	54.85 ± 0.04^b^
Zinc (mg/100 g)	2.68 ± 0.03^a^	3.44 ± 0.03^b^
Dietary fiber		
Soluble dietary fiber (g/100 g)	0.71 ± 0.03^a^	0.81 ± 0.03^b^
Insoluble dietary fiber (g/100 g)	12.89 ± 0.06^a^	13.25 ± 0.05^b^
Total dietary fiber (g/100 g)	13.60 ± 0.08^a^	14.06 ± 0.04^b^
Total starch and starch nutritional fractions		
Rapidly digestible starch (%)	19.76 ± 0.23^a^	21.15 ± 0.31^b^
Slowly digestible starch (%)	14.1 ± 0.10^a^	18.87 ± 0.25^b^
Resistant starch (g/100 g)	24.39 ± 0.61^a^	27.01 ± 0.59^b^
Total starch (g/100 g)	58.25 ± 0.47^a^	67.03 ± 1.08^b^
Predicted glycemic index and glycemic load		
Predicted glycemic index	52.57 ± 0.18^b^	48.94 ± 0.21^a^
Glycemic load	31.68 ± 0.16^b^	28.83 ± 0.46^a^

The superscripts mean that these values are significantly different from each other.

### 4.2. Organoleptic scores of the developed products

The sensory characteristics of the developed value added-products are presented in Table 3. Among them, *papad* obtained the highest overall acceptability score of 8.46 ± 0.50 followed by rusk (8.04 ± 0.49), *kheer* (8.00 ± 0.50), *sattu* (8.00 ± 0.41), *pinni* (7.98 ± 0.47), cookies (7.86 ± 0.40), vegetable *dalia* (7.82 ± 0.43), and millet bar (7.80 ± 0.52). *Sattu* and *papad* were found to have higher overall acceptability scores than the traditional products, while other developed products showed a lower range of scores; however, all of them were found to be acceptable, scoring more than 7.5 on the hedonic scale. The sensory variables, including color, appearance, texture, taste and flour of rusk, sattu and papad, were found to be comparable with their control counterparts, while, other products obtained a lower score for these variables. However, the prepared products were still found acceptable by the panel. No differences (*p* > 0.05) were found among the sensory characteristics (color, appearance, texture, taste, flavor, and overall acceptability) between the Foxtail millet- and cereal-based products.

**Table 3:** Organoleptic evaluation of the products prepared from Foxtail millet.

Products		Color	Appearance	Texture	Taste	Flavor	Overall acceptability
Rusk	C	7.70 ± 0.48^a^	7.70 ± 0.48^a^	7.50 ± 0.70^a^	7.70 ± 0.48^a^	7.70 ± 0.48^a^	7.66 ± 0.50^a^
	E1	8.00 ± 0.81^a^	8.00 ± 1.05^a^	8.10 ± 0.31^a^	8.00 ± 0.47^a^	8.10 ± 0.56^a^	8.04 ± 0.49^a^
*Kheer*	C	8.20 ± 0.42^a^	8.20 ± 0.42^a^	8.20 ± 0.42^a^	8.20 ± 0.42^a^	8.20 ± 0.42^a^	8.20 ± 0.42^a^
	E1	8.20 ± 0.63^a^	8.10 ± 0.56^a^	7.80 ± 0.42^a^	8.00 ± 0.66^a^	7.90 ± 0.56^a^	8.00 ± 0.50^a^
*Pinni*	C	8.20 ± 0.63^a^	8.00 ± 0.47^a^	8.00 ± 0.47^a^	7.90 ± 0.56^a^	8.00 ± 0.47^a^	8.02 ± 0.39^a^
	E1	8.00 ± 0.47^a^	7.80 ± 0.42^a^	8.10 ± 0.56^a^	8.00 ± 0.66^a^	8.00 ± 0.66^a^	7.98 ± 0.47^a^
*Sattu*	C	7.80 ± 0.63^a^	7.80 ± 0.63^a^	7.70 ± 0.67^a^	7.70 ± 0.67^a^	7.70 ± 0.67^a^	7.74 ± 0.64^a^
	E1	8.00 ± 0.47^a^	8.00 ± 0.47^a^	8.00 ± 0.47^a^	8.00 ± 0.47^a^	8.00 ± 0.47^a^	8.00 ± 0.41^a^
Vegetable *Dalia*	C	8.10 ± 0.32^a^	8.10 ± 0.31^a^	8.20 ± 0.42^a^	8.20 ± 0.42^a^	8.20 ± 0.42^a^	8.16 ± 0.35^a^
	E1	7.80 ± 0.42^a^	7.80 ± 0.42^a^	7.90 ± 0.56^a^	7.80 ± 0.63^a^	7.80 ± 0.63^a^	7.82 ± 0.43^a^
Cookies	C	8.30 ± 0.49^b^	8.10 ± 0.56^a^	8.10 ± 0.56^a^	8.30 ± 0.48^a^	8.30 ± 0.48^a^	8.22 ± 0.45^a^
	E1	7.70 ± 0.82^a^	7.60 ± 0.70^a^	7.90 ± 0.31^a^	8.10 ± 0.31^a^	8.00 ± 0.47^a^	7.86 ± 0.40^a^
*Papad*	C	8.30 ± 0.49^a^	8.30 ± 0.49^a^	8.40 ± 0.51^a^	8.40 ± 0.51^a^	8.40 ± 0.51^a^	8.36 ± 0.48^a^
	E1	8.50 ± 0.52^a^	8.40 ± 0.51^a^	8.40 ± 0.51^a^	8.50 ± 0.52^a^	8.50 ± 0.52^a^	8.46 ± 0.50^a^
Millet bar	E1	7.80 ± 0.42	7.90 ± 0.56	7.60 ± 0.70	7.90 ± 0.56	7.80 ± 0.63	7.80 ± 0.52

C- Control (prepared from traditional cereal); E1-Foxtail millet F2 Values are expressed as Mean ± SD of triplicates.

Values having different alphabetical superscripts represent significant difference (*p* ≤ 0.05) among the various sensory characteristics.

### 4.3. Nutritional composition of the developed value-added products

**Figure 3:**
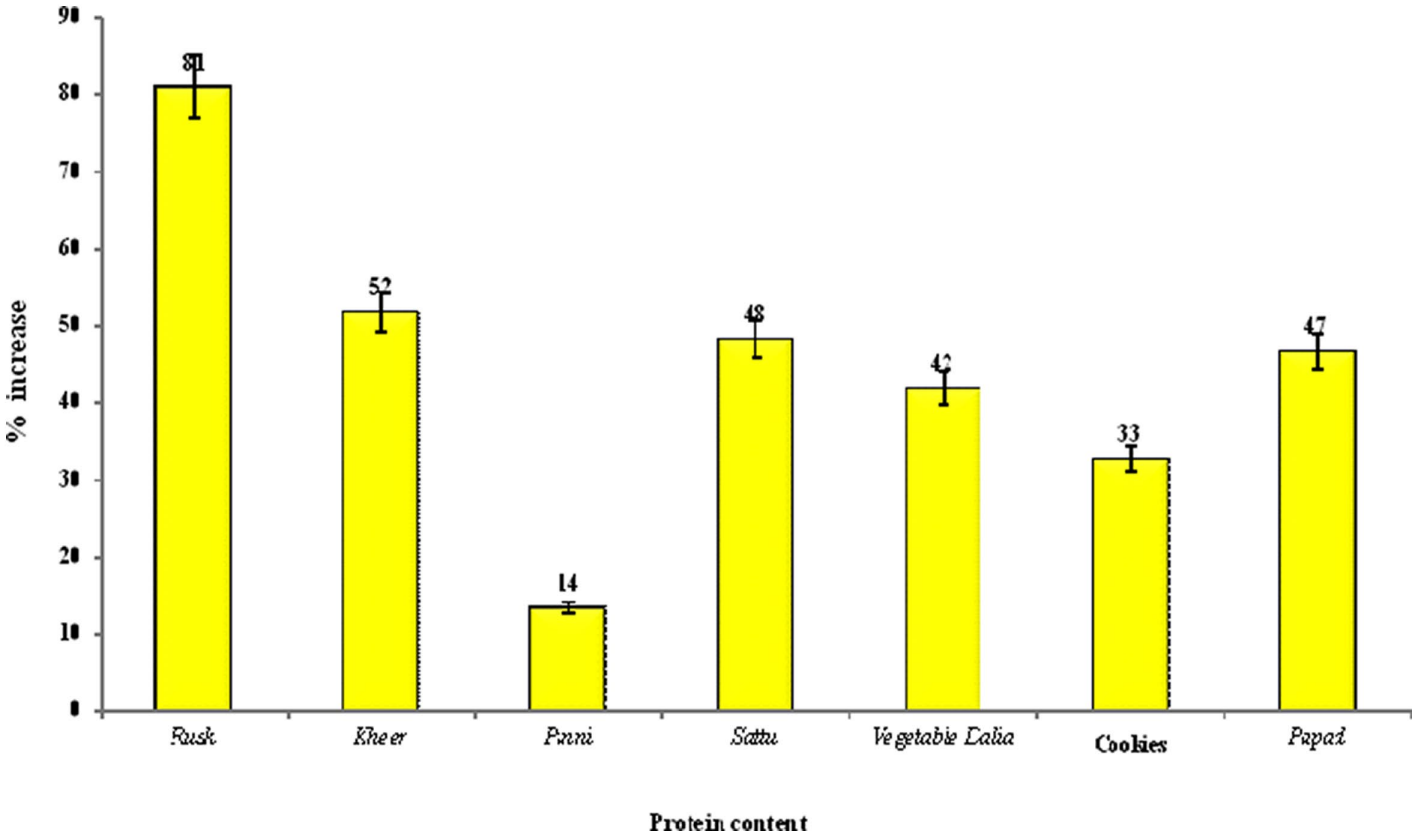
Percent increase in the protein content of the food products prepared from Foxtail millet with respect to control group.

The crude protein content in the value-added products of Foxtail millet ranged from 10.98 to 16.10 g/100 g on dry weight basis. The protein content of the developed food products was significantly (*p* ≤ 0.05) higher than the control products, which can be due to the greater amount of protein found in millets than traditional cereal (wheat/rice). The highest content of crude protein was found in the Foxtail millet *Kheer* (16.01 g/100 g), which can be attributed to the basic ingredient milk used in the recipe. The increase in crude protein content of the developed products in comparison to control is shown in Figure 3. The increase in the protein content of the developed products ranged from 14 to 81%, with a maximum increase of 81% in millet *rusk*, in comparison to the refined wheat flour *rusk*.

The fat content of the developed products was found in the range of 3.00 ± 0.16 to 30.45 ± 0.17 g/100 g with maximum content in *pinni* (30.45 ± 0.17 g/100 g), while the minimum was found in *sattu* (3.00 ± 0.16 g/100 g). A significant difference (*p* ≤ 0.05) was observed in the crude fat content of the products between the control and the experimental group. The highest fiber content was found in *sattu* (3.86 ± 0.15 g/100 g).

All the Foxtail millet products showed a higher amount of minerals (Fe, Zn, and Ca) than their control counterparts. The iron, zinc and calcium content of the developed products ranged from 3.03 ± 0.04 to 4.21 ± 0.04, 1.37 ± 0.08 to 3.16 ± 0.07, and 53.30 ± 0.16 to 157.81 ± 0.28 mg/100 g, respectively, with the corresponding values of 0.97 ± 0.07 to 4.07 ± 0.02, 0.5 ± 0.07 to 1.89 ± 0.04 and 10.60 ± 0.18 to 110.57 ± 0.33 mg/100 g, in the traditional cereal based products. The highest iron content was found in *sattu* (4.21 ± 0.04 mg/100 g), whereas the highest zinc and calcium contents were observed in vegetable *dalia* (3.16 ± 0.07 mg/100 g) and *kheer* (157.81 ± 0.28 mg/100 g), respectively. On the other hand, *kheer* was found to have the lowest iron content (3.03 ± 0.04 mg/100 g), while cookies showed the lowest content of zinc (1.37 ± 0.08 mg/100 g) and calcium (53.30 ± 0.16 mg/100 g).

The soluble, insoluble and total dietary fiber of the developed products are presented in Table 4A. A significant (*p* ≤ 0.05) difference was found among the soluble, insoluble and total dietary fiber of Foxtail millet and traditional cereal-based products, with the respective values ranging from 0.41 ± 0.02 to 0.80 ± 0.02, 7.27 ± 0.16 to 12.67 ± 0.25 and 7.72 ± 0.12 to 13.47 ± 0.23 g/100 g in the Foxtail millet-based products, while the corresponding values in the control group were 0.40 ± 0.02 to 0.79 ± 0.02, 3.26 ± 0.06 to 9.62 ± 0.11 and 3.82 ± 0.06 to 10.32 ± 0.06 g/100 g, respectively. The developed products had a higher total dietary fiber content, with the highest amount of all fractions of dietary fiber found in *sattu* (13.47 ± 0.23 g/100 g), followed by bar (12.83 ± 0.06 g/100 g).

**Table 4A:** Nutritional composition of the value added products.

Products		Moisture (%)	Protein (g/100 g)	Crude fat (g/100 g)	Crude fiber (g/100 g)	Ash (g/100 g)	CHO (g/100 g)	Iron (mg/10 0 g)	Zinc (mg/10 0 g)	Calcium (mg/100 g)
Rusk	C	2.64 ± 0.08^b^	7.33 ± 0.25^a^	3.26 ± 0.18^a^	0.87 ± 0.04^a^	2.10 ± 0.2^a^	83.79 ± 0.43^b^	1.71 ± 0.05^a^	0.98 ± 0.40^b^	40.78 ± 0.56^a^
	E1	2.29 ± 0.07^a^	13.27 ± 0.46^b^	4.12 ± 0.17^b^	2.68 ± 0.15^b^	2.26 ± 0.15^a^	74.53 ± 0.95^a^	3.61 ± 0.70^b^	1.70 ± 0.01^a^	63.69 ± 0.39^b^
*Kheer*	C	68.51 ± 1.10^b^	10.60 ± 0.04^a^	4.45 ± 0.37^a^	0.81 ± 0.03^a^	1.53 ± 0.2^a^	14.08 ± 1.67^a^	1.84 ± 0.03^a^	1.67 ± 0.03^a^	110.57 ± 0.33^a^
	E1	61.38 ± 1.19^a^	16.10 ± 0.88^b^	5.66 ± 0.32^b^	3.09 ± 0.04^b^	3.16 ± 0.32^a^	8.81 ± 1.25^a^	3.03 ± 0.04^b^	2.16 ± 0.06^b^	157.81 ± 0.28^b^
*Pinni*	C	4.02 ± 0.31^b^	9.67 ± 0.19^a^	27.76 ± 0.31^a^	1.77 ± 0.10^a^	3.10 ± 0.2^b^	53.67 ± 0.41^a^	1.35 ± 0.03^a^	1.89 ± 0.04^a^	59.58 ± 0.62^a^
	E1	2.71 ± 0.10^a^	10.98 ± 0.07^b^	30.45 ± 0.17^b^	3.69 ± 0.67^b^	2.33 ± 0.2^a^	51.03 ± 2.28^a^	3.96 ± 0.03^b^	2.06 ± 0.09^b^	93.74 ± 0.40^b^
*Sattu*	C	3.59 ± 0.17^a^	8.04 ± 0.23^a^	2.20 ± 0.19^a^	1.54 ± 0.08^a^	1.83 ± 0.2^a^	82.78 ± 0.31^b^	4.07 ± 0.02^a^	1.01 ± 0.02^a^	28.54 ± 0.08^a^
	E1	3.94 ± 0.74^a^	11.93 ± 0.69^b^	3.00 ± 0.16^a^	3.86 ± 0.15^b^	3.60 ± 0.26^b^	72.65 ± 0.85^a^	4.21 ± 0.04^b^	1.99 ± 0.03^b^	57.53 ± 0.34^b^
Vegetable	C	72.49 ± 0.94^b^	8.36 ± 0.76^a^	3.30 ± 0.18^a^	1.84 ± 0.04^a^	2.13 ± 0.25^a^	8.86 ± 0.83^a^	3.01 ± 0.02^a^	1.83 ± 0.09^a^	36.43 ± 0.45^a^
*Dalia*	E1	65.45 ± 0.08^a^	11.87 ± 0.11^b^	4.72 ± 0.08^b^	3.46 ± 0.07^b^	4.60 ± 0.17^a^	10.89 ± 0.89^b^	3.67 ± 0.04^b^	3.16 ± 0.07^b^	58.25 ± 0.10^b^
Cookies	C	2.34 ± 0.13^b^	8.46 ± 0.66^a^	5.70 ± 0.08^a^	0.87 ± 0.04^a^	1.33 ± 0.15^a^	81.92 ± 0.59^b^	1.35 ± 0.05^a^	0.89 ± 0.61^b^	20.44 ± 0.31^a^
	E1	2.78 ± 0.05^a^	11.23 ± 0.50^b^	5.90 ± 0.12^b^	2.41 ± 0.06^b^	1.46 ± 0.20^a^	76.22 ± 0.70^a^	3.32 ± 0.03^b^	1.37 ± 0.08^a^	53.30 ± 0.16^b^
*Papad*	C	1.82 ± 0.09^a^	8.71 ± 0.44^a^	6.18 ± 0.19^a^	0.89 ± 0.03^a^	0.87 ± 0.06^a^	81.51 ± 0.18^b^	0.97 ± 0.07^a^	0.5 ± 0.07^a^	10.60 ± 0.18^a^
	E1	2.12 ± 0.05^b^	12.78 ± 0.73^b^	8.37 ± 0.09^b^	1.41 ± 0.04^b^	1.26 ± 0.2^b^	71.04 ± 0.72^a^	3.66 ± 0.02^b^	1.04 ± 0.02^b^	57.38 ± 0.22^b^
Millet bar	E1	4.64 ± 0.09	14.20 ± 0.56	4.71 ± 0.26	3.64 ± 0.04	2.60 ± 0.1	70.19 ± 0.60	3.60 ± 0.02	1.77 ± 0.05	56.63 ± 0.33

C- Control; E1-Foxtail millet F2.

Values are expressed as Mean ± SD of triplicates.

Values having different alphabetical superscripts represent significant difference (*p* ≤ 0.05) among the products in the control and the experimental group.

Rapidly digestible starch was found to be significantly (*p* ≤ 0.05) higher in the control group than the developed products, among which, the maximum rapidly digestible starch content was observed in *papad* (32.26%), while the minimum content was found in *sattu* (19.9%). The maximum amount of slowly digestible starch was seen in *kheer* (21.12%).

The resistant starch content of the developed products ranged from 13.67 ± 0.29 to 22.61 ± 0.69 g/100 g, with the corresponding values of 7.65 ± 0.29 to 16.35 ± 0.41 g/100 g found in the traditional cereal based products. The highest content of resistant starch was found in the bar (22.61 ± 0.69) g/100 g followed by vegetable *dalia* (17.16 ± 0.61), *kheer* (16.91 ± 0.34), *papad* (15.76 ± 0.46), cookies (15.32 ± 0.90), *sattu* (14.08 ± 0.34), *pinni* (13.67 ± 0.29), and rusk (13.97 ± 0.72) g/100 g.

**Figure 4:**
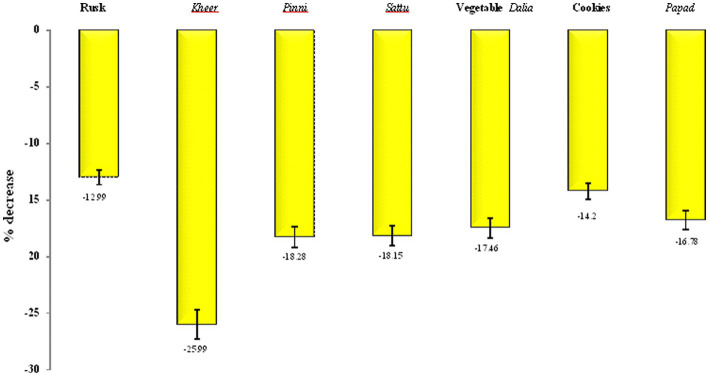
Percent decrease in the predicted glycemic index of the food products prepared from Foxtail millet with respect to control group.

The predicted glycemic index of the developed products ranged from 46.12 ± 0.12 to 57.55 ± 0.34, while the control group products had a predicted glycemic index ranging from 60.23 ± 0.30 to 72.23 ± 0.07. The lowest predicted glycemic index was found in bar (46.12 ± 0.12), followed by *sattu* (49.30 ± 0.19), cookies (53.05 ± 0.44), *kheer* (53.46 ± 0.30), *papad* (54.76 ± 0.14), rusk (56.27 ± 0.10), vegetable *dalia* (57.13 ± 0.19), and *pinni* (57.55 ± 0.34). The decrease in predicted glycemic index of the Foxtail millet products compared to the cereal based products is shown in Figure 4. The maximum percent decrease in GI was found in Foxtail millet *pinni* (18.28%), *papad* (16.78%), cookies (14.2%) and rusk (12.99%). Hence, the glycemic index of the products prepared from millets decreased in comparison to control group, in a range of 8–28%, proving that Foxtail millet products had a low GI in comparison to wheat and rice products (Table 4B).

**Table 4B:** Nutritional composition of the value added products.

Products		Soluble dietary fiber (g/100 g)	Insoluble dietary fiber (g/100 g)	Total dietary fiber (g/100 g)	Rapidly digestible starch (%)	Slowly digestible starch (%)	Resistant starch (g/100 g)	Total starch (g/100 g)	Predicted glycemic index	Glycemic load
Rusk	C	0.56 ± 0.0 _3_a	3.26 ± 0.06^a^	3.82 ± 0.06^a^	29.94	17.90	7.65 ± 0.29^a^	55.49 ± 0.34^b^	64.36 ± 0.30^b^	53.92 ± 0.05^b^
	E 1	0.46 ± 0.0 _4_a	7.27 ± 0.1 _6_b	7.72 ± 0.1 _2_b	25.40	11.55	13.97 ± 0.72^b^	50.92 ± 1.09^a^	56.27 ± 0.10^a^	40.25 ± 0.30^a^
*Kheer*	C	0.65 ± 0.0 _3_a	3.98 ± 0.09^a^	4.64 ± 0.11^a^	30.61	21.89	16.35 ± 0.41^a^	68.85 ± 0.94^b^	72.23 ± 0.07^b^	7.56 ± 1.13^b^
	E 1	0.70 ± 0.0 _4_b	8.93 ± 0.3 _2_b	9.63 ± 0.3 _3_b	24.89	21.12	16.91 ± 0.34^a^	62.92 ± 1.18^a^	53.46 ± 0.30^a^	4.69 ± 0.70^a^
*Pinni*	C	0.69 ± 0.0 _3_a	7.80 ± 0.14^a^	8.50 ± 0.12^a^	38.42	8.82	8.86 ± 0.80^a^	56.10 ± 1.39^b^	70.42 ± 0.26^b^	37.80 ± 0.43^b^
	E 1	0.70 ± 0.0 _5_a	12.09 ± 0.12^b^	12.79 ± 0.13^b^	29.36	7.47	13.67 ± 0.29^b^	50.50 ± 1.09^a^	57.55 ± 0.34^a^	29.37 ± 1.43^a^
*Sattu*	C	0.71 ± 0.0 _6_a	9.62 ± 0.11^a^	10.34 ± 0.15^a^	22.24	23.84	9.52 ± 0.36^a^	55.60 ± 1.02^b^	60.23 ± 0.30^b^	49.86 ± 0.14^b^
	E 1	0.80 ± 0.0 _2_a	12.67 ± 0.25^b^	13.47 ± 0.23^b^	19.19	18.6	14.08 ± 0.34^b^	51.87 ± 0.38^a^	49.30 ± 0.19^a^	35.81 ± 0.39^a^
Vegetable *Dalia*	C	0.40 ± 0.0 _2_a	6.56 ± 0.24^a^	7.21 ± 0.50^a^	32.07	11.85	10.52 ± 0.52^a^	54.44 ± 1.23^a^	69.93 ± 0.19^b^	6.19 ± 0.18^a^
	E 1	0.41 ± 0.0 _2_a	11.25 ± 0.09^b^	11.64 ± 0.10^b^	29.83	5.06	17.16 ± 0.61^b^	52.05 ± 0.63^a^	57.13 ± 0.19^a^	6.28 ± 0.53^a^
Cookies	C	0.79 ± 0.0 _2_b	4.92 ± 0.10^a^	4.71 ± 0.08^a^	30.06	19.83	9.93 ± 0.50^a^	59.82 ± 0.83^a^	63.75 ± 0.08^b^	44.03 ± 0.24^b^
	E 1	0.64 ± 0.0 _4_a	7.41 ± 0.2 _0_b	8.05 ± 0.1 _8_b	25.00	14.22	15.32 ± 0.90^b^	54.54 ± 0.68^b^	53.05 ± 0.44^a^	37.23 ± 0.15^a^
*Papad*	C	0.54 ± 0.0 _2_a	3.39 ± 0.10^a^	4.24 ± 0.10^a^	33.17	5.94	13.05 ± 0.23^a^	52.16 ± 0.76^a^	65.80 ± 0.23^b^	53.64 ± 0.29^b^
	E 1	0.71 ± 0.0 _3_a	11.57 ± 0.39^b^	12.28 ± 0.35^b^	32.26	7.06	15.76 ± 0.46^b^	55.08 ± 0.76^b^	54.76 ± 0.14^a^	41.74 ± 0.36^a^
Millet bar	E 1	0.72 ± 0.03	12.11 ± 0.11	12.83 ± 0.06	23.21	10.83	22.61 ± 0.69	56.65 ± 0.97	46.12 ± 0.12	35.15 ± 0.36

C- Control; E1-Foxtail millet F2.

Values are expressed as Mean ± SD of triplicates.

Values having different alphabetical superscripts represent significant difference (*p* ≤ 0.05) among the products in the control and the experimental group.

Also, glycemic load (GL) is directly proportional to glycemic index; hence the products with low glycemic index depicted a low glycemic load, whereas the products in the control group, which had higher glycemic index were found to have a higher glycemic load. The glycemic load of the developed Foxtail millet products ranged from 4.69 ± 0.70 to 41.74 ± 0.36, with the lowest glycemic load observed in millet bar (35.15 ± 0.36), whereas the highest glycemic load was found in *papad* (41.74 ± 0.36). The glycemic load of *kheer* was low (4.69 ± 0.70), while it was found to have a glycemic index of 53.46 ± 0.30.

**Figure 5:**
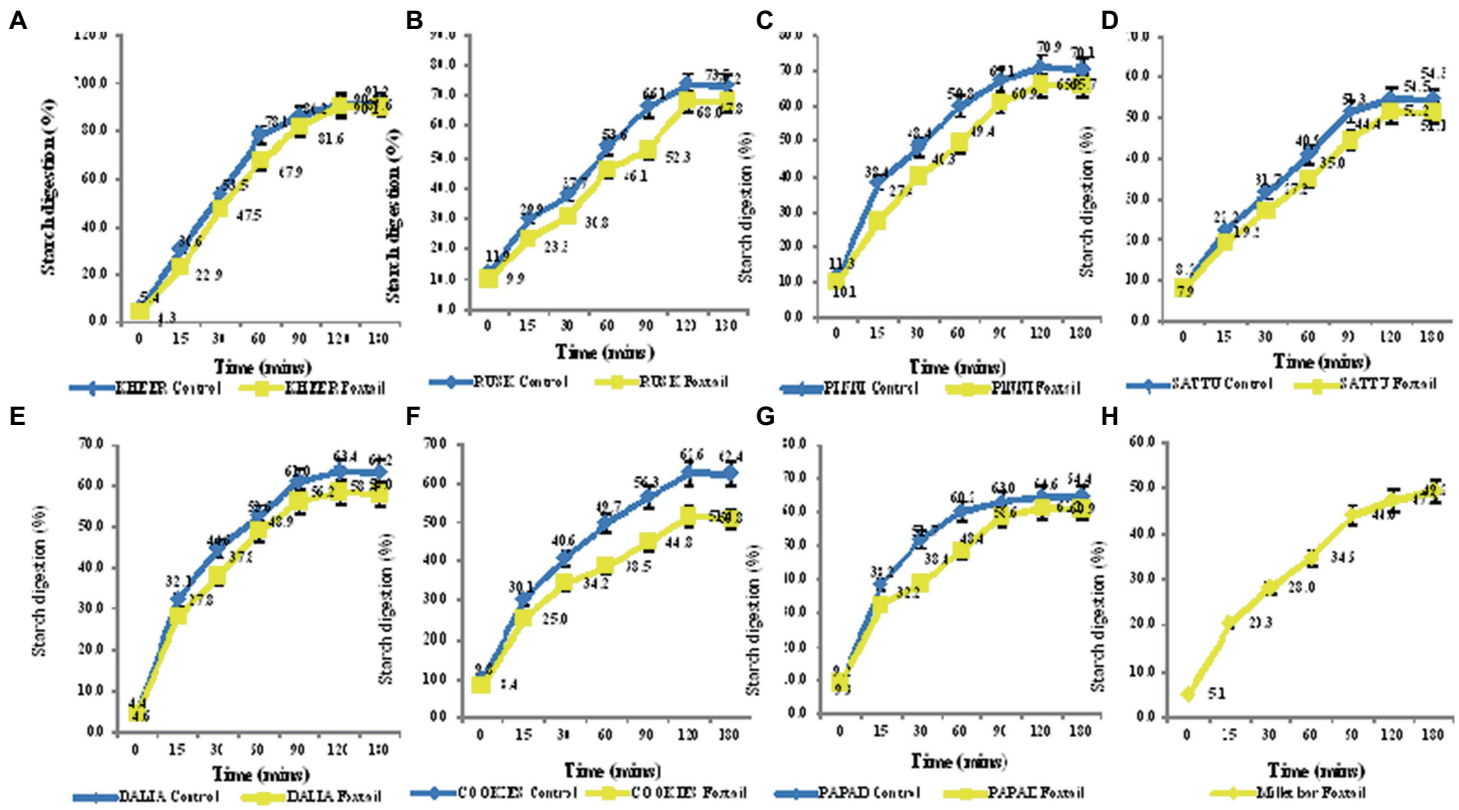
**(A–H)** *In vitro* starch digestion rate of value added products prepared from Foxtail millet and the control group.

It was observed that at the end of 180 min of *in vitro* digestion, more amount of the products from the control group were digested than the millet-based products. The slowest *in vitro* starch digestion rate at the end of 180 min of digestion was seen in bar (51.5%). On the other hand, Foxtail millet *kheer* was found to have a higher rate to the tune of 87.1%, while 91.2% of the starch in rice-based *kheer* was found to be hydrolysed at the end of 180 min. Majority of the millet-based products were found to have a digestion rate ranging from 50 to 60%, while their control counterparts had 54.3–91.2% digestion rate at the end of 180 min (Figure 5).

## 5. Discussion

The present study was conducted with an objective to explore the possibility of replacement of traditional cereals, mainly wheat and rice, with millets. The millets contain low glycemic index than other cereal grains which can be due to the higher dietary fiber content and resistant starch which have the potential to slow down the absorption of glucose (35). Foxtail millet had a slower starch digestion rate than wheat, which can be due to the fact that millets have higher amount of protein than other cereals due to which there is a reduced gelatinization and enzyme permeability, resulting from the protein-starch gel’s encapsulation of starch globules. Also, a higher content of fats in millets lead to prevention of amylase entry by a lipophilic amylo-lipid inclusion complex (36). It was reported that the *in vitro* starch digestion rate ranged between 22.29 and 35.52% in nine varieties of Pearl millet (37). Therefore, various research studies mentioned above proved better nutritional profile of foxtail millet than wheat.

Organoleptic properties are the aspects of food, water, or other substances that a person perceives through their senses, which include taste, sight, smell, and touch. The purpose of the sensory evaluation is to describe the product. The value-added products were developed by replacing the cereal (wheat/rice) with millet using the standardized recipe. Those recipes which are already liked by the population and fit well in the Indian cuisine were selected and replaced with millets. The incorporation of millets was initiated at a level of 10% and all the recipes were found to be acceptable even at 100% replacement.

The value-added products namely rusk*, kheer, pinni, sattu,* vegetable *dalia, papad*, cookies and millet bar were developed and compared with the products prepared from traditional cereal (wheat for *rusk*, cookies, vegatable *dalia* and *pinni*, rice for *papad* and kheer and barley for *sattu*), which were considered as control for their organoleptic scores. The products *viz.,* r*usk, kheer, pinni,* vegetable *dalia,* cookies and formulated from Foxtail millet in the present study obtained overall acceptability scores comparable to the control dishes. It was in line with the study, where Foxtail millet was used to make food items such as *laddu*, vegetable biryani, and halwa and received the highest scores for overall acceptability, color, appearance, texture, and flavor than their control counterparts (38). In another study, various combinations of Foxtail millet flour were used to make *chakli* mix. The *chakli* made entirely of Foxtail millet flour was found to have the best overall appearance, flavor, texture, and acceptability (39). Foxtail millet was used to make *laddu*, peanut *chutney*, *panjeeri, kheer,* cutlet, and *chakli,* among other things. The products were scored numerically by 38 semi-trained panelists. *Laddu, kheer,* and *panjeeri* were found to be highly acceptable, followed by peanut *chutney*, whereas *chakli* was not. Thus, Foxtail millet can be easily included in the preparation of various traditional recipes without compromising their sensory qualities (40). Many other research studies also showed higher acceptability of Foxtail millet products like cookies (41), *papad* (42) and millet-based drinks (43) than their traditionally prepared counterparts. The higher acceptability of millet based products can be due to the fact that during roasting and baking, browning leads to an acceptable, pleasant odor. Frying increases the palatability of the product. These cooking techniques employed might have resulted in overcoming the bitterness of the millet grains (44).

The prepared products also had a higher protein, fiber, and fat content in comparison to the control counterparts. Addition of different ingredients like milk during preparation of recipes might have increased these nutritional parameters. Literature also reports that products prepared from millets are rich sources of macronutrients (45). The difference in the fat content of the products can be due to the addition of ingredients in the recipe and the type of cooking method used. The highest fat content in *pinni* may be attributed to the addition of fat and flax seeds, whereas the lowest fat in *sattu* can be due to the roasting of grains, while preparing *sattu* powder. Roasting leads to denaturation of lipolytic enzymes, which may be responsible for loss of fat after processing. A reduction of 51% in the fat content was found after roasting of products (46). *Laddu* and *halwa* were developed from Foxtail and Barnyard millet and a crude fat content of 25.63 and 25.01% in *laddu* and 9.60 and 9.29% in *halwa*, respectively, were reported (38). Similarly, pearl millet *papad* (80% pearl millet, 20% rice flour) were prepared and a fat content of 5.55% was reported, which is in line with the present findings (47). The high fiber content in *sattu* can be attributed to the roasting of grains, as during roasting there is a partial degradation of cellulose and hemicellulose that decreases the insoluble dietary fiber content (48). The least fiber content observed in *papad* can be due to the cooking method employed. *Papad* was steamed prior to drying and deep- frying, which resulted in the breakdown of lignin bonds, breaking the cellulosic structure of the fiber (49).

The highest iron content in *sattu* can be attributed to the addition of jaggery, having a high iron content of 10–13 mg/100 g as reported by (50). The high iron content of *Sattu* can also be attributed to the cooking technique used, where in roasting in a regular pan led to higher iron retention as reported by (51). Similarly, the increased zinc content of vegetable *dalia* can be due to the fact that millet *dalia* was soaked prior to the cooking process and soaking of millets boosts the *in vitro* solubility of the zinc by 2–23% and also decreases phytic acid content (52). The maximum calcium content in *kheer* may be due to the higher content of calcium present in milk than the other ingredients used in various recipes. Similar results were reported by (53) in which finger millet-based *sheera* (prepared using Finger millet and milk) had a calcium content of 149.2 mg/100 g.

The total starch content of the products prepared from traditional cereals was significantly (*p* ≤ 0.05) higher than the Foxtail millet products, which ranged from 50.50 ± 1.09 to 62.92 ± 1.18 g/100 g, with the highest content found in the Foxtail millet *kheer*. The highest total starch content in *kheer* was due to the addition of sugar, which increased the total carbohydrate content and hence, the total starch content. The more rapidly digestible starch in *papad* can be due to frying of *papad.* Frying affects starch digestibility in terms of structural damage which leads to an increase in rapidly digestible starch (54). Slowly digestible starch content was observed in vegetable *dalia* (5.06%) which can be due to pressure cooking that results in maximum gelatinization, that increases the content of rapidly digestible starch and reducing slowly digestible starch (55). The maximum amount of slowly digestible starch in *Kheer* may be because of its refrigeration prior to serving, which led to a new structure formation converting the RDS to SDS, hence, increasing the slowly digestible starch and reducing the rapidly digestible starch (56).

A significant difference (*p* ≤ 0.05) was seen in the resistant starch content of the products prepared from Foxtail millet and the traditional cereal except in the case of *kheer*. The temperature at which food is kept after cooking affects its resistant starch content (57). The refrigeration results in increased resistant starch content, and, therefore, it should be consumed after sufficient refrigeration so as to gain maximum health benefits.

Resistant starch content of the products is greatly affected by the cooking technique. The cooking methods like pressure cooking, boiling, microwave cooking and baking lead to a decrease in its concentration, while roasting can potentially retain the resistant starch content (58), which is in line with the findings of the present study. Moreover, the cooking process can affect the starch digestion rate. The structure of starch in foods changes at higher temperatures (52). Roasting is a relatively dry thermal process that hinders gelatinization of starch during processing and may result in decreasing starch digestion rate (58). The highest resistant starch in bar can be attributed to the fact that Millet bar was prepared with roasting, which is one of the efficient ways to retain it in millets (58). Foods high in resistant starch are favorable for the people suffering from diabetes and cardiovascular diseases (57), as this starch is not rapidly digested in the body and is fermented in the colon by microorganisms (59). The millet grain is superior to other cereals in terms of both the quality and amount of dietary fiber and resistant starch. These ingredients help to prolong the sense of fullness and avoid constipation. Furthermore, by binding to toxins in the stomach, they protect the colon mucosa from cancer (57).

The glycemic index of the product depends upon its total available carbohydrate content and the type of starch present in it. Also, the high concentration of polyphenols and other bioactive components lead to reduction in the rate of fat absorption and sugar release resulting in lesser glycemic index (13). Moreover, higher amount of resistant starch, protein and dietary fiber lowers the predicted glycemic index of the food product. The millet bar and *sattu* had the lowest glycemic index, which can be due to their higher resistant starch and higher dietary fiber content. In the current investigation, millet-based products showed a lower GI than the wheat/rice-based products. The lower glycemic load of *kheer* can be due to its high moisture content as high moisture content of the food results in a lower available carbohydrate content of the food product, resulting in a lower glycemic load. It has been reported that watermelon and high-fat ice cream have glycemic index of 72 and 37, respectively, whereas both were found to have same glycemic load of 4, which was attributed to high moisture content of watermelon that led to lesser available carbohydrate leading to a lower glycemic load (60).

Similar to the findings of the present study, biscuits were prepared from barnyard millet and refined wheat flour in the combination of 45:55 ratio and their glycemic index was reported as 68 (41), whereas barnyard millet *burfi* prepared by adding 43% millet had a glycemic index of 45 (61). The GI of food products made from 100% proso millet was 50–65, compared to 70–80 for refined corn and wheat-based products. Millets have a lower glycemic index (GI) than wheat, rice, and barley, making them an ideal food for people with type 2 diabetes and cardiovascular disease (CVD) (62). The protective role of millets against hyperglycemia by replacing rice-based dosa with Foxtail millet dosa for people with type 2 diabetes was demonstrated by switching from a meal made of rice to one made of millet, which decreased postprandial blood glucose levels in T2DM patients (63).

The slow digestion rate of Foxtail millet-based products can be due to the presence of higher amounts of protein and fat than wheat-rice products. It was reported that during processing, starch can interact with proteins, which is thought to affect starch digestibility (64). Denatured protein may stick to starch granules, preventing enzyme absorption. Also the gel that results from the hydrophilic interaction of protein and starch can enclose globules and even influence the gelatinization process. The impact of protein on *in vitro* starch digestion rate and the *in vitro* glycemic response was studied and an increase in both due to absence of protein starch complex was reported (65). Similarly, the presence of high lipid content reduces the *in vitro* starch digestion rate by forming a lipophilic amylo-lipid inclusion complex, which refrains the entry of amylase (66). The higher amount of dietary fiber in millets also reduces the *in vitro* starch digestion rate, by binding the water and reducing gelatinization (67).

## 6. Conclusion

Foxtail millet-based food products were developed by 100% replacement of wheat/rice/barley. The developed food products obtained acceptable sensory scores and were found to be at par against the traditional products. The foxtail millet-based products contained higher levels of protein, dietary fiber, resistant starch, and had lower glycemic index and starch digestion rate than wheat/ rice/ barley-based products. The developed Foxtail millet products can be categorized as foods with a medium glycemic index and can be used as therapeutic foods for diabetics. *Kheer* prepared from Foxtail millet also had high fiber and calcium contents, which can be useful for osteoporosis and can be consumed as a healthy snack. The formulated Foxtail millet products can be helpful in the prevention of non- communicable diseases like diabetes and obesity, owing to its high fiber, resistant starch and lower glycemic index.

## Data availability statement

The raw data supporting the conclusions of this article will be made available by the authors, without undue reservation.

## Author contributions

LA: investigation, methodology, and writing—original draft. RA: supervision and project administration. RA and ID: conceptualization, resources, and final draft. OG and PK: formal analysis and final draft. All authors contributed to the article and approved the submitted version.

## Conflict of interest

The authors declare that the research was conducted in the absence of any commercial or financial relationships that could be construed as a potential conflict of interest.

## Publisher’s note

All claims expressed in this article are solely those of the authors and do not necessarily represent those of their affiliated organizations, or those of the publisher, the editors and the reviewers. Any product that may be evaluated in this article, or claim that may be made by its manufacturer, is not guaranteed or endorsed by the publisher.
